# Soap Making

**Published:** 1888-11

**Authors:** 


### SOAP MAKING.

The accurate history of the manufacture of soap stretches back to the factories built at Marseilles, when there was an apparent recognition of the principles of saponification. Neither then nor until centuries later, however, was there any desire to understand what the principle was, and for many years every effort to wrest the secret from chemistry and make soap boiling an art was fought by the manufacturer and workman. "The factories at Marseilles had around them all the materials necessary for soap making," says a recent English work upon this art. "Thc olive tree, the fruit of which yields a fixed oil in great abundance, flourished in the south of France, while the shores of the Mediterranean yielded an ample supply of maritime plants from which crude soda was obtained by calcination. As the time progressed Italy furnished olive oil, while Spain contributed crude soda, or barilla." The gradual development of the art while extremely interesting to the chemist, is of no special interest to the general reader. Leblanc's discovery of a process for the manufacture of soda from common salt, Chevaeul's explanation of the nature of the reaction which takes place when fatty substances are treated with boiling solutions of caustic alkali, gave an exactness to the manufacture of soap such as it had never before had ; but it was a long time before the boilers would avail themselves of the aid of these men of science. Steam succeeded the ordinary fire, and the list of fatty substances used in soap making, grew and grew, until there are now a dozen of them forming the base of soap, with over 100 entering into the composition of different kinds of soap to a greater or less degree—*The Kitchen.*

